# Supplementary material for: Viral pathogens hitchhike with insect sperm for paternal transmission
Source: Nat Commun. 2019 Feb 27;10:955. doi: 10.1038/s41467-019-08860-4 (PMC6393494; doi:10.1038/s41467-019-08860-4)
Supplement: Supplementary file 1 — Supplementary Information [file 41467_2019_8860_MOESM1_ESM.pdf]

Supplementary Information

Viral pathogens hitchhike with insect sperm for paternal transmission

Mao et al.

Supplementary Material and Methods

Insect and virus. *R. dorsalis* females or males were collected from Xingning, Guangdong, China and propagated on non-infected rice seedlings in cages at 25 ± 1°C with 75 ± 5% relative humidity and 16-h light/8-h dark. To establish the nonviruliferous leafhopper population, the original leafhoppers (1st generation) collected from virus-free rice fields in Guandong Province were kept in glass tubes containing one rice seedling individually to lay eggs and then were detected for RGDV using RT-PCR. The offspring (2nd generation) produced by RGDV-negative parent were reared individually in glass tubes until eclosion, and one male and one female leafhopper were picked out to mate for 4 days. After mating, the males of the 2nd generation were collected for RGDV detection and the females were left to lay eggs for 8 days and then collected for RGDV detection. The 3rd generation offspring produced by RGDV-negative parents were picked out and treated in the same way with the 2nd generation. The 4th generation leafhopper were used as nonviruliferous population. To obtain a viruliferous leafhopper population, the 2nd- or 3rd- instar nymph of *R. dorsalis* were fed on diseased rice plants for 1 day and then transferred to healthy rice seedlings and the viruliferous rate is up to 80%[1]. RGDV isolates were also collected from Xingning, Guangdong, China and maintained on rice plants via transmission by *R. dorsalis*[2]. The *N. cincticeps* population was collected in Xingning, Guangdong, China and maintained on rice plants. The *P. alienus* population was provided by Dr. Xifeng Wang from Chinese Academy of Agricultural Sciences.

Field monitoring of viruliferous status of *R. dorsalis*. The frequency of viruliferous individuals of *R. dorsalis* in population from Xingning was examined every 45 days during the winter (November to February) each year from 2013 to 2017. For each survey, at least 1,000 leafhoppers were collected from 10 rice fields (667 m^2 per field) using a chessboard sampling

method. The virus was detected using RT-PCR assay, and the viruliferous rate was calculated using the weighted average algorithm based on the data from each rice field. The viruliferous rates in 50 males and in 50 females of *R. dorsalis* from each of the 10 rice fields was also determined using RT-PCR assay during the 2015-2016 overwintering season.

Investigation of RGDV-infection in *Alopecurus aequalis*. To monitor RGDV infection rate in the weed *Alopecurus aequalis* in Xingning, around 80 plants were randomly collected from 5 rice fields each year from 2015-2018 for virus detection by RT-PCR assay. Alternately, 30 healthy weed seedlings were collected in the field each year from 2015-2018 and exposed to the laboratory-reared viruliferous *R. dorsalis*. Each seedling was exposed to 3 viruliferous adults for a 5-day inoculation access period and then collected for RGDV detection depending on both visible disease symptoms and RT-PCR assay at 21 days after inoculation.

Successive paternal or maternal transmission of RGDV. Two crossing treatments (V^{-}♀ × V^{+}♂ or V^{+}♀ × V^{-}♂) were conducted, and the offspring produced by the females in each of the two treatments (1st generation) were reared in individual test tubes each with one rice seedling. The males or females of the 1st generation were collected after eclosion and each individual was allowed to mate with a V^{-} virgin female or V^{-} male adult for 4 days. After mating, the males of the 1st generation were collected for RGDV detection, and the females were left to lay eggs for 8 days and then collected for RGDV detection. The 2nd and 3rd offspring were treated in the same way with the 1st generation. Ten replicates were conducted for each generation in each of the two treatments.

Fitness measurement. The effects of RGDV infection on the longevity of female and male *R. dorsalis* adults were evaluated by placing one 1st instar nymph of viruliferous *R. dorsalis* on

one non-infected rice seedling in a glass tube, and one nonviruliferous insect was placed in a glass tube under the same conditions as control. One hundred nymphs were conducted for each group. The longevity of each leafhopper was monitored at 12-h interval until the end of adult life. Each developmental stage was recorded individually. All nymphs and adults were assayed for virus by RT-PCR assay following their death. The entire experiment was replicated three times. The effect of RGDV on mating behavior of an adult male *R. dorsalis* was examined in terms of the number of females the male copulated with in a given time interval for observation. Each virgin V^+ or V^- male adult was reared with 10 V^- virgin female adults in a glass tube containing one rice seedling for 24, 48 and 72 h respectively. Each female was transferred to another glass tube containing rice seedlings for oviposition after copulation, and the hatching of nymphs in each tube was inspected as an indicator of successful mating. Five replicates were performed for each of the three treatments, and the entire experiment was replicated three times.

The effects of paternal or maternal transmission on offspring development were evaluated in terms of embryonic development. Four crossing treatments were conducted as described for the crossing experiments above. At 7 days postoviposition, the eggs were collected from the seedlings and the number of eggs laid by the female was recorded. Eggs with red eyespots were identified as an indicator of embryonic development to evaluate the effect of maternal or paternal transmission on egg hatching using the equation of egg hatching rate = number of eggs with eyespots/total number of eggs. For the four crossing treatments (V^-♀ × V^-♂, V^+♀ × V^-♂, V^-♀ × V^+♂ and V^+♀ × V^+♂), 11, 10, 9 and 10 pairs were tested for mating and oviposition, and 998, 736, 951 and 840 eggs were observed for hatching, respectively. In addition, 100 eggs with red eyespots were randomly sampled from the eggs deposited by females in each of the four crossing treatments, and the length of each egg was measured using an anatomical lens and imaging equipment (Nikon SMZ18).

The effects of paternal or maternal transmission on fecundity were evaluated in terms of the number of eggs produced by individual females in each of the four crossing treatments (V^-♀ × V^-♂, V^+♀ × V^-♂, V^-♀ × $^+$♂ and V^+♀ × V^+♂) in a 4-day-oviposition period. For the four treatments, 16, 16, 11 and 10 replicates were done respectively.

The effects of paternal or maternal virus transmission on the sex ratio of offspring were evaluated. The offspring produced by the females in each of the four treatments (V^-♀ × V^-♂, V^+♀ × V^-♂, V^-♀ × $^+$♂ and V^+♀ × V^+♂) were reared in individual test tubes each with one rice seedling until eclosion and the sex ratio was distinguished and recorded. To compare the effects of vertical transmission manner on viral transmission ability of offspring, individual adult offspring produced in each of the four treatments were exposed to a new healthy rice seedling in individual tubes for a 2-day inoculation access feeding and then picked out for RGDV detection using RT-PCR assay. The rice plants were tested for RGDV infection depending on both visible symptoms and RT-PCR assay at 21 days after inoculation.

Transmission of RGDV by females acquired virus from V^+ male. To detect viral transmission ability of female *R. dorsalis* obtained RGDV from V^+ males through venereal transmission, virgin V^- female adults were mated one on one with V^+ male adults in individual glass tubes for 3 days. Males were then collected and confirmed for RGDV-positive by RT-PCR assay. Individual females at 3, 6 and 10 days after mating with the V^+ males were exposed to a new healthy rice seedling in individual tubes for a 3-day inoculation access period and then collected for RGDV detection using RT-PCR assay. The rice plants were tested for RGDV presence by RT-PCR assay and the presence of visible disease symptoms at 21 days after inoculation. Ten plants were performed each time and the entire experiment was replicated three times.

Preparation of antibodies. To generate mouse polyclonal antibodies against HSPG, the domain III fragment (bp 1,513-5,028) of the HSPG gene of *R. dorsalis* was amplified and inserted into vector pH4. The recombinant plasmid was used to transform *E. coli* strain *Rosetta* to express the targeted protein, which was then injected into mice to produce antibodies. To test the specificity of antibodies against HSPG, we extracted total proteins from male *R. dorsalis* and rice plants. Samples were separated by SDS-PAGE and probed with antibodies against HSPG.

Polyclonal antisera against the minor outer capsid protein P2 and the major capsid protein P8 of RGDV were injected into rabbits as described above. RGDV virions were purified from infected rice plants and then injected into rabbits to produce antibodies. IgGs were purified from the antiserum sample using a protein A-Sepharose affinity column and then conjugated directly to fluorescein isothiocyanate (FITC) or rhodamine according to the manufacturer's instructions (Invitrogen).

To determine whether antibodies against HSPG specifically recognized sperm HSPG *in vivo*, the midgut, testes and smears of mature sperm from nonviruliferous leafhoppers were fixed, stained with HSPG-specific antibodies conjugated to FITC (HSPG-FITC) (0.5 μg/μl) and then processed for confocal microscopy. To determine the function of HSPG in viral binding to the sperm head surface, the mature sperm smears obtained from viruliferous male leafhoppers were fixed, stained with HSPG-FITC, virus-rhodamine and DAPI, and then processed for confocal microscopy.

Neutralizing RGDV-sperm binding. In neutralization experiments to test the direct interaction between RGDV P8 and HSPG, mature sperms were excised from nonviruliferous leafhoppers that had been pre-incubated for 30 min with either 3% BSA, pre-immune serum (as controls) or antibodies against HSPG (0.5 μg/μl). *In vitro* RGDV-sperm binding experiments

were then performed as described above. Alternatively, purified RGDV particles were premixed for 10 min with purified protein that was encoded by the HSPG Domain III fragment and expressed in *E. coli* strain *Rosetta*, and then incubated with live sperm smears for 30 min. Sperm samples were then stained and processed for confocal microscopy visualization.

Detection of HSPG expression in *R. dorsalis*. To compare the protein and gene expression levels of HSPG in V^+ and V^- male *R. dorsalis*, or in V^- male and female *R. dorsalis*, we excised the reproductive systems from V^+ males, V^- males or V^- females at 5 days after eclosion. Total protein was extracted from 50 male reproductive systems, and HSPG levels were analyzed by Western blot assay with HSPG-specific IgG. Total RNAs were extracted using Trizol reagent (Invitrogen) from 30 male or female reproductive systems, and then the transcript levels of HSPG gene were quantified by relative RT-qPCR assay with the SYBR Green PCR MasterMix Kit (Promega) in a Mastercycler Reaplex4 real-time PCR system (Eppendorf) in accordance with the manufacturer's instructions. The relative levels of gene expression were normalized to a housekeeping gene EF1 and estimated by the $2^{-\triangle\triangle Ct}$ (cycle threshold) method. The experiment was replicated three times, and a pool of 30 insects was used for each replicate. To compare the mRNA and protein levels of HSPG in the male reproductive system and the remaining insect tissues of *R. dorsalis,* male adults at 5 days after eclosion were collected and divided into two parts, reproductive system and the remaining tissues. Total proteins and total RNAs were extracted as described above.

Paternal transmission of RGDV by *N. cincticeps.* To ascertain the paternal transmission of RGDV by minor leafhopper vector *N. cincticeps,* a series of experiments were conducted. To compare the viruliferous rates of individuals of *R. dorsalis* and *N. cincticeps* in populations, 25 males and 25 females were collected from 5 rice fields in Xingning and detected for

RGDV positive using RT-PCR assay. To compare viral acquisition efficiencies of *R. dorsalis* and *N. cincticeps,* fifty 3rd- or 4th- instar nymphs were reared on RGDV-infected rice plants for 3 days, and then transferred onto non-infected rice seedlings for 10 days. All insects were collected for RT-PCR assay using RGDV specific primers. Three replicates were conducted. To detect the vertical transmission of RGDV in *N. cincticeps*, two crossing treatments (V^+♀ × V^-♂ and V^-♀ × V^+♂) were conducted with the laboratory-reared colony as described above. The viruliferous leafhoppers were obtained through microinjection with purified RGDV virions (0.01μg/μl). In each treatment, one newly emerged adult female was crossed with one newly emerged adult male, and the number of eggs detected in the five independent experiments was as follows: (i) V^+♀ × V^-♂, $n_1 = 13$, $n_2 = 13$, $n_3 = 18$, $n_4 = 20$, $n_5 = 16$; (ii) V^-♀ × V^+♂, $n_1 = 13$, $n_2 = 13$, $n_3 = 18$, $n_4 = 20$, $n_5 = 16$. For visualizing viral association with the sperms in *R. dorsalis* and *P. alienus*, second-instar nymphs of *N. cincticeps* and *P. alienus* were microinjected with purified RGDV virions (0.01μg/μl) and the mature sperms were excised from the seminal vesicles of male adults, immunolabeled with virus-rhodamine and observed as described above.

Supplementary Figures

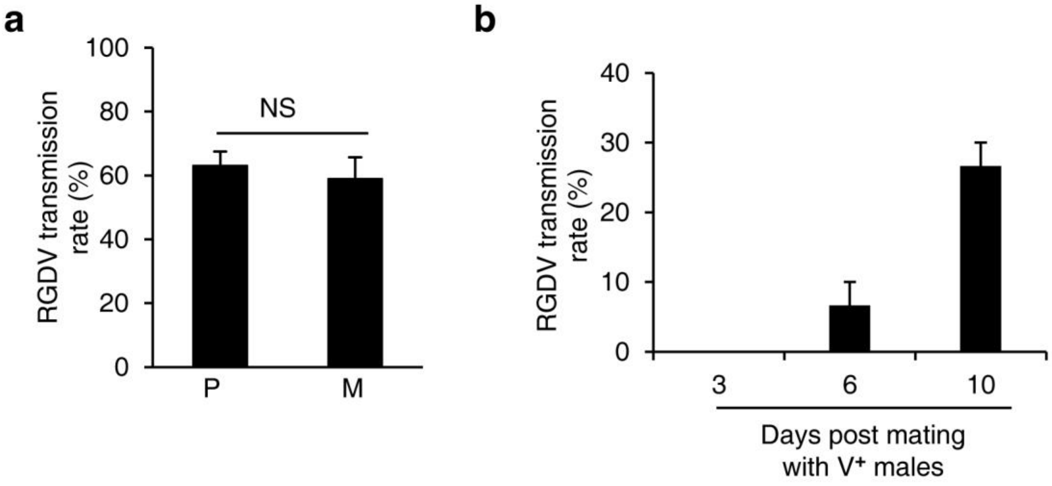

Supplementary Figure 1 - Transmission of RGDV obtained via paternal, maternal or venereal transmission manners. **a** Transmission rates of RGDV by viruliferous offspring derived from viruliferous male (paternal transmission, P) or female (maternal transmission, M) parent to rice plants. **b** Transmission rates of RGDV by female adult *R. dorsalis* after mated with V^+ males to rice plants. Data are means (±SD) from 3 independent experiments. The significance of any differences was tested using Student's t-test (two-tailed). NS, no significant difference.

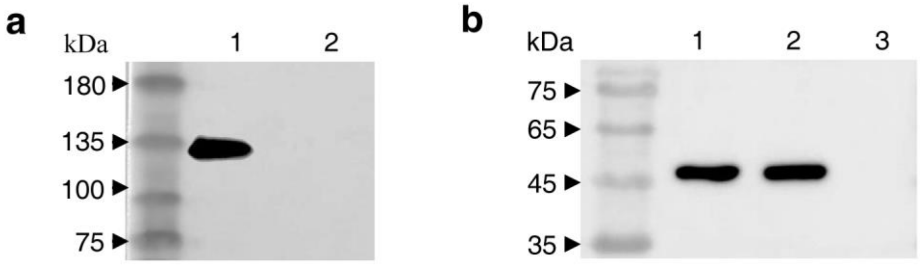

Supplementary Figure 2 - Specificity of antibodies against P2 and P8 of RGDV. **a** Western blot assay for the detection of P2 of RGDV by antibodies against P2. Lanes 1-2, proteins extracted from viruliferous and nonviruliferous *R. dorsalis*, respectively. **b** Western blot assay for the detection of P8 of RGDV by antibodies against P8. Lanes 1-3, proteins extracted from viruliferous (1 and 2) and nonviruliferous (3) *R. dorsalis*, respectively.

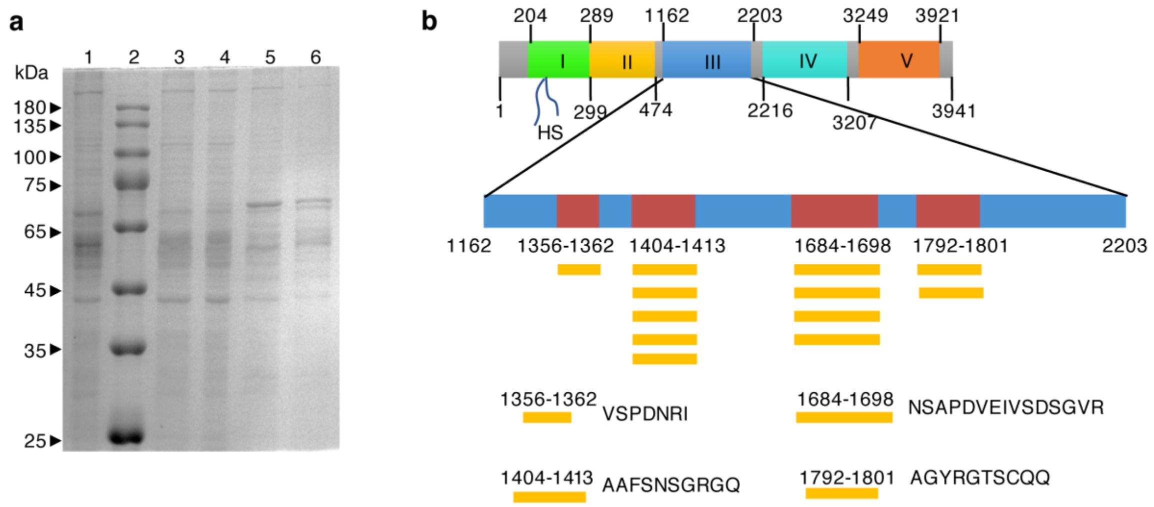

Supplementary Figure 3 - Screening for sperm proteins that interact with RGDV P8. **a** Detection of sperm proteins using 1-DE electrophoresis obtained through GST pull-down assay. Lanes: 1, proteins extracted from sperms of *R. dorsalis*; 2, protein marker; 3 and 4, eluted sperm proteins bound to GST-conjugated Sepharose beads; 5 and 6, eluted sperm proteins bound to GST-RGDV P8-conjugated Sepharose beads, and the eluted sperm proteins were then sent for mass spectrometry analysis. **b** Peptides targeting the HSPG of *R. dorsalis* were obtained by mass spectrometry analysis. Schematic representation of *R. dorsalis* HSPG gene containing five different domains. The 12 identified peptides with four different types totally mapped to the domain III of HSPG.

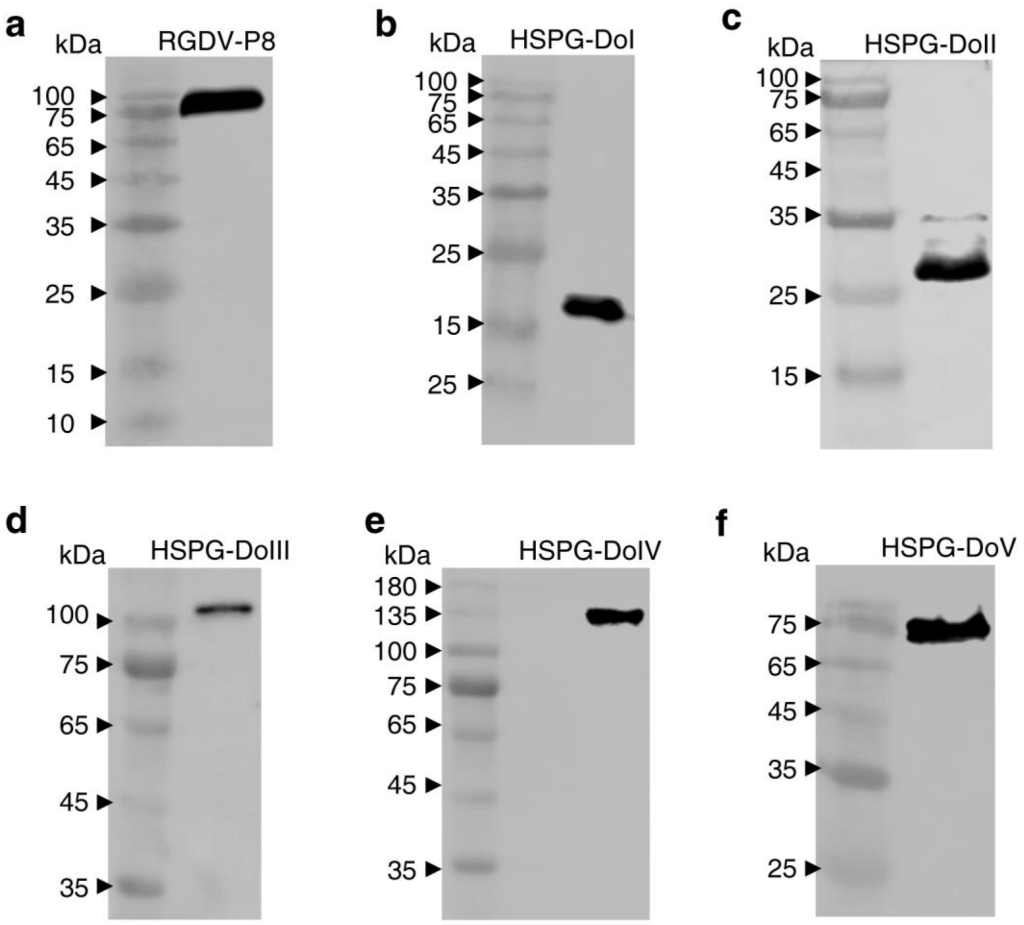

Supplementary Figure 4 - Detection of RGDV P8 and HSPG domains I-V in yeast. **a** Detection of RGDV P8 using antibodies against LexA encoded by pBT3-STE. **b-f** Detection of domains I-V of HSPG using antobodies against HA Tag encoded by pPR3-N.

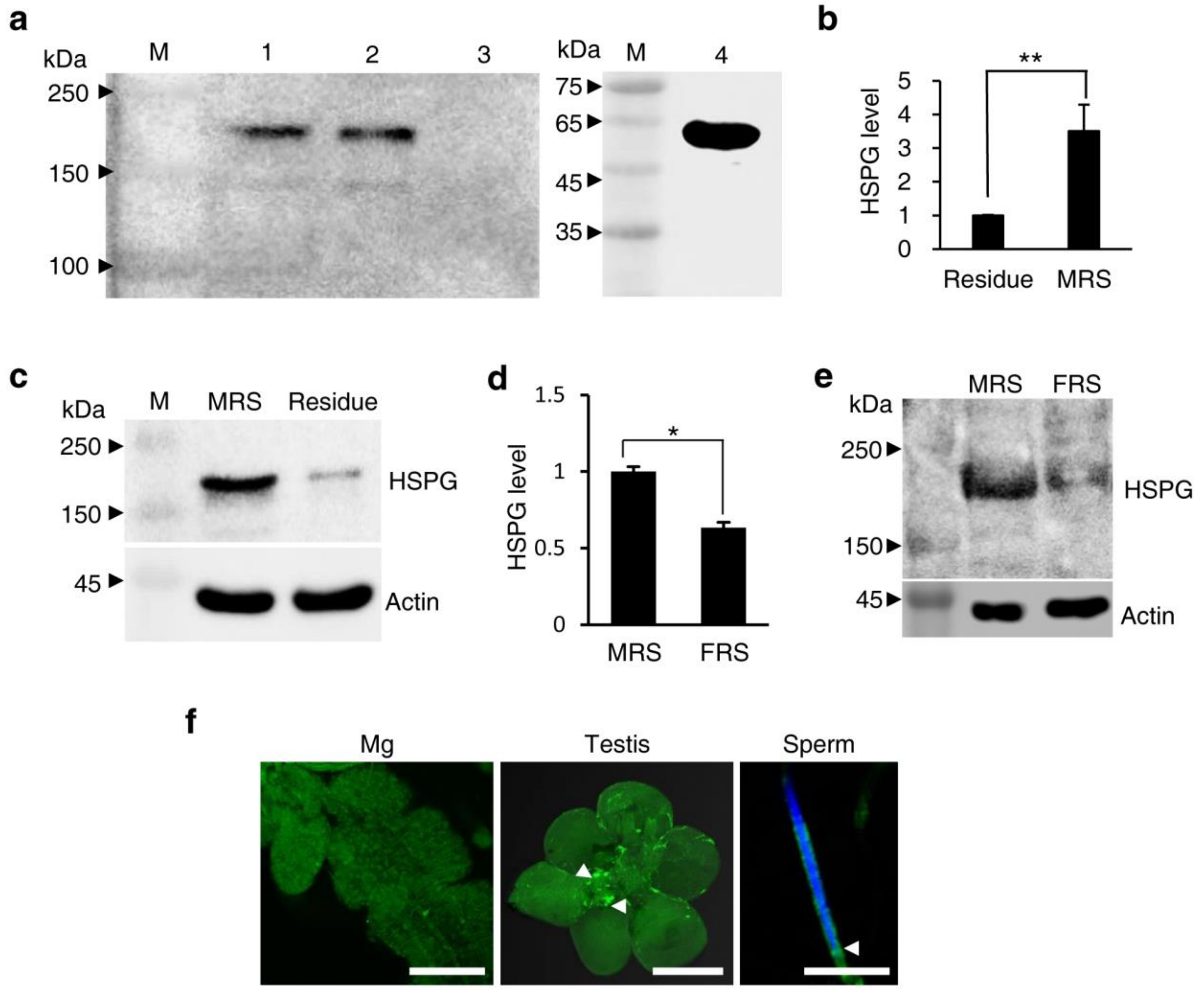

Supplementary Figure 5 - Levels of HSPG in different tissues of *R. dorsalis*. **a** Specificity of antibodies against HSPG. Samples were separated by SDS-PAGE and detected with antibodies against HSPG. Lanes: M, protein marker; 1, 2 and 3 are proteins extracted from male *R. dorsalis* adults (1 and 2) and rice plants (3), respectively; 4 is HSPG domain III expressed by *E. coli*. **b** Transcript levels of HSPG gene in male reproductive system (MRS) and the rest of the insect body (Residue), as determined by RT-qPCR assay. Data are means (±SD) from 3 independent experiments. The significance of any differences was tested using Student's *t*-test. $^{**}P < 0.01$. **c** Levels of HSPG in male reproductive system (MRS) and in the rest of the insect body (Residue), as determined by Western blot assay using HSPG-specific IgG. Insect actin was detected with actin-specific IgG as a control. **d** Transcript levels of HSPG gene in male reproductive system (MRS) and female reproductive system (FRS), as

determined by RT-qPCR assay. Data are means (±SD) from 3 independent experiments. The significance of any differences was tested using Student's *t*-test (two-tailed). $^{*}P < 0.05$. **e** Levels of HSPG in male reproductive system (MRS) and female reproductive system (FRS), as determined by Western blot assay using HSPG-specific IgG. Insect actin was detected with actin-specific IgG as a control. **f** Immunofluorescence microscopy showing distribution of HSPG in the midgut (Mg), testis and sperm excised from seminal vesicle. Tissues were stained with HSPG-FITC (green) or DAPI (blue). White triangles indicate HSPG signals. Bars: panel Mg and Testis, 50 μm; panel Sperm, 5 μm. All images are representative of at least 3 replicates.

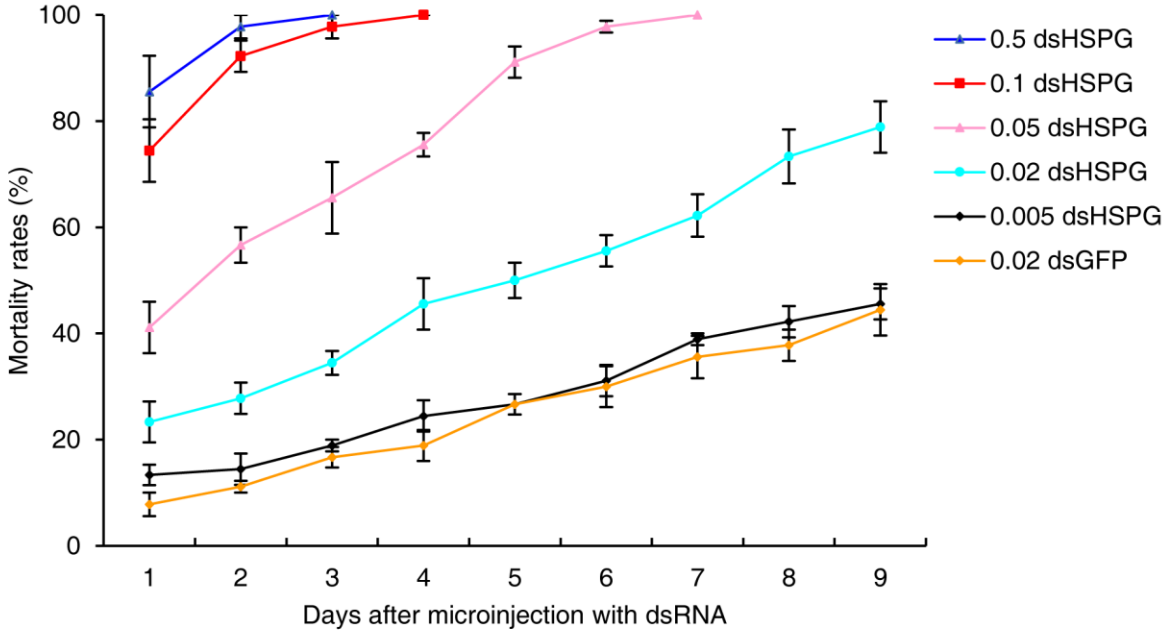

Supplementary Figure 6 - Mortality of V$^+$ male *R. dorsalis* treated with dsGFP or dsHSPG. V$^+$ male leafhoppers were microinjected with dsHSPG at 0.5, 0.1, 0.05, 0.02, 0.005 or 0.02 μg/μL, and the leafhopper mortality was measured. The mortality of adults injected with dsGFP was used as the control.

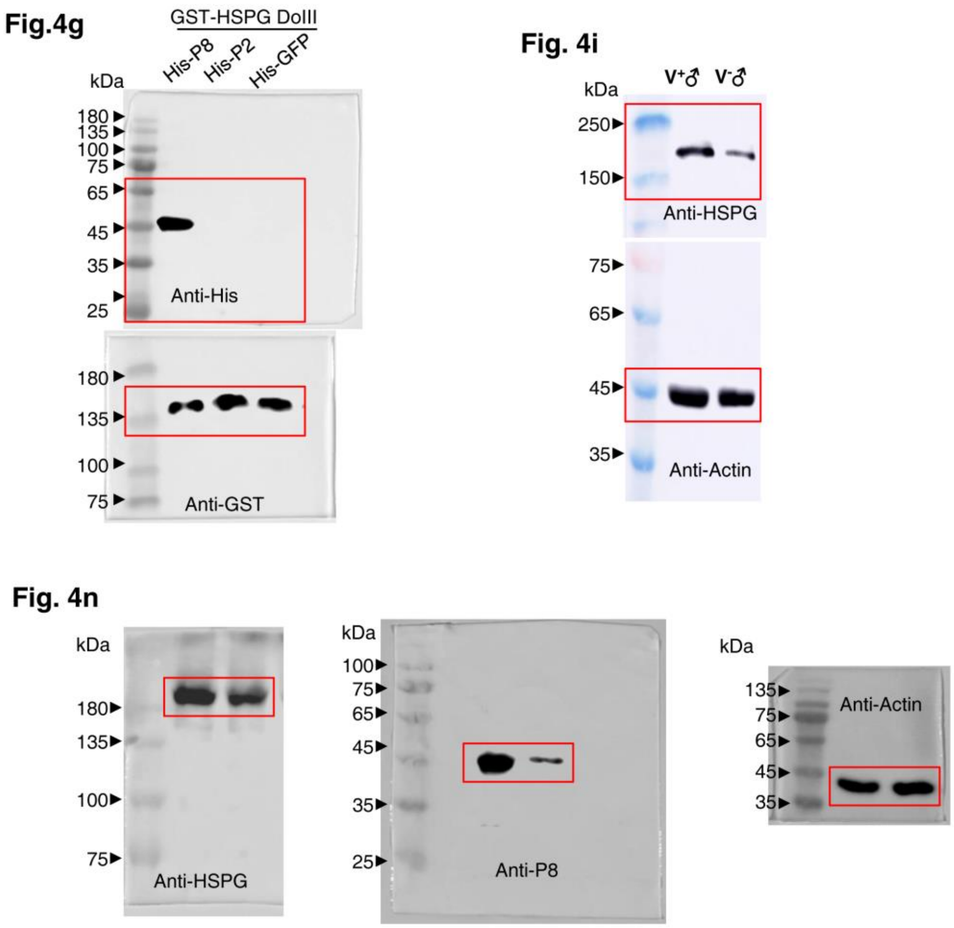

Original images for immunoblots shown in Figs.4g, 4i and 4n. The molecular weight markers and the antibodies used are indicated on the images.

Fig. S2

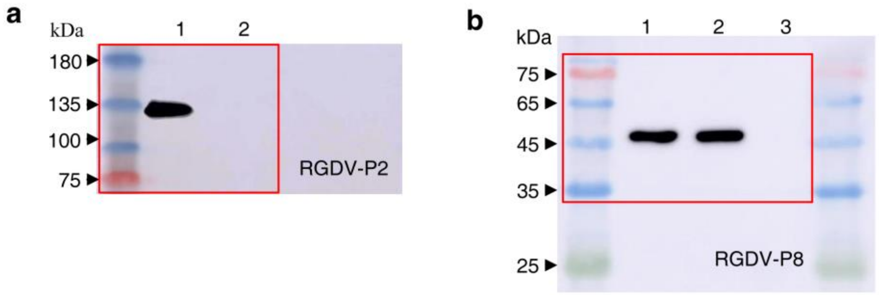

Original images for immunoblots shown in Supplementary Figs.2a and 2b. The molecular weight markers and the antibodies used are indicated on the images.

Fig. S3a

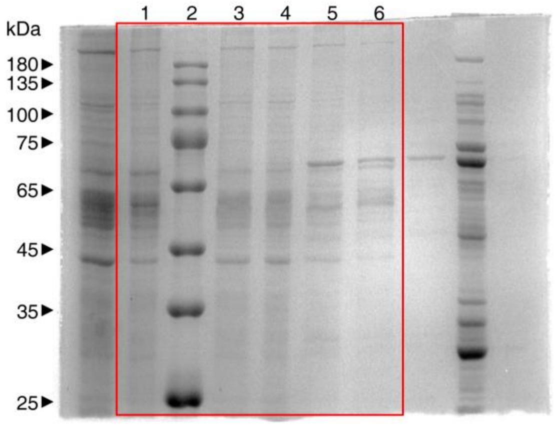

Original image for the immunoblot shown in Supplementary Fig.3a. The molecular weight markers and the antibodies used are indicated on the image.

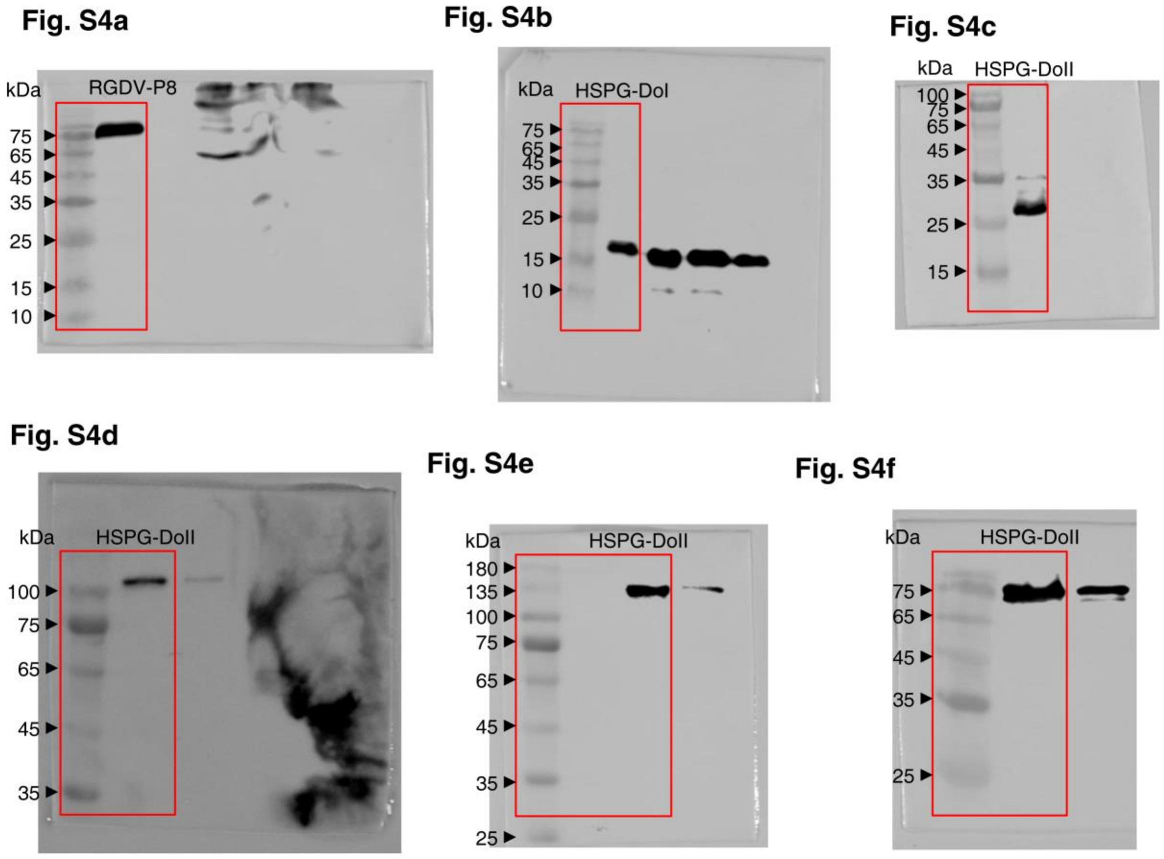

Original images for immunoblots shown in Supplementary Figs.4a-f. The molecular weight markers and the antibodies used are indicated on the images.

Fig. S5a

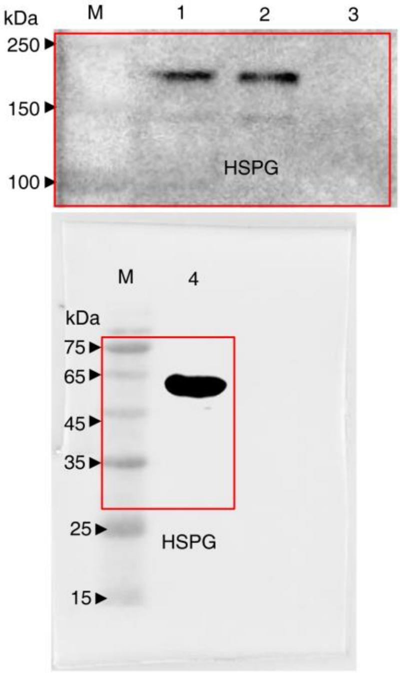

Fig. S5c

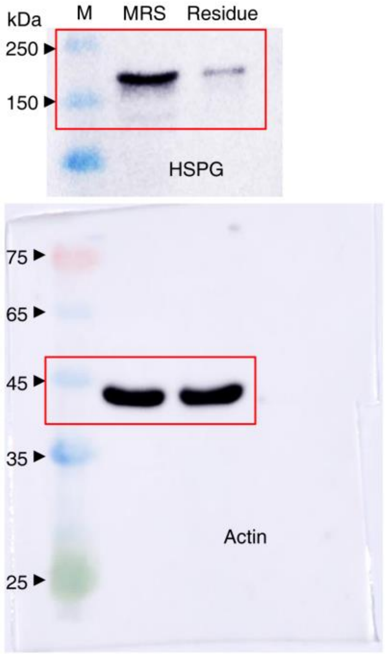

Fig. S5e

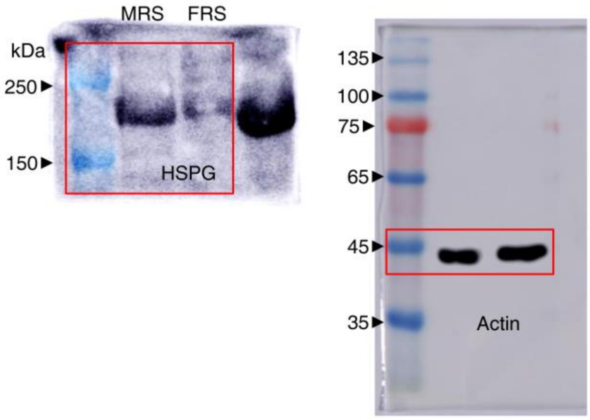

Original images for immunoblots shown in Supplementary Figs.5a, 5c and 5e. The molecular weight markers and the antibodies used are indicated on the images.

Supplementary Tables

Supplementary Table 1 - Viral infection of the weed *A. aequalis*.

Sample year	No. of weed plants infected with RGDV/no. of plants observed
2015 in the field	0/87
2016 in the field	0/68
2017 in the field	0/80
2015 under laboratory condition	1/30
2016 under laboratory condition	2/30
2017 under laboratory condition	1/30

Supplementary Table 2 - Distribution of RGDV in V^+ males at different days after eclosion.

Tissues	No. of insects positive for RGDV (*n* = 30)		
	3 days	7 days	9 days
Testis	20	22	26
Accessory gland	20	22	26
Seminal vesicle	19	22	25
Intestine	24	25	28

Supplementary Table 3 - Distribution of RGDV in reproductive system of V^{-} females after mating with V^{+} males.

Tissues	No. of insects positive for RGDV (n = 30)			
	3 days	6 days	10 days	12 days
Spermatheca	23	25	25	24
Oviduct	0	6	25	24
Terminal filament	0	5	23	24
Pedicel	0	7	24	24
Oocyte	0	0	0	1
Intestine	0	4	19	20

Supplementary Table 4 - List of oligonucleotide primers used in this study.

Oligonucleotide	Assay	Sequence (5'-3')
RD_EF1_F	qPCR	CAGTGAGAGCCGTTTTGAG
RD_EF1_R	qPCR	AGGGCATCTTGTCAGAGGGC
RGDV_P8_F	qPCR	TGACCTTCATCGTCTCTGAGTCCGA
RGDV_P8_R	qPCR	CGTTACCATTAACCGCGTTCACCTG
RD_HSPG_F	qPCR	TGGACACAGACGACGGACTTGA
RD_HSPG_R	qPCR	AAGGCAGGTTCGGTGACGGTAA
RD_HSPG_53_F	cloning	CCCTGGCGCTGCTTCCAACACCTTT
RD_HSPG_1287_R	cloning	GTCCCGGCACTCAAACTCATCCGGC
RD_HSPG_1123_F	cloning	TCACTCTCTACGACGGACTGCGCCG
RD_HSPG_2210_R	cloning	GCACAGTCCTCCTCGTCGCTGGAGT
RD_HSPG_2082_F	cloning	TTGTCCGAGTGCCCAGACCTGCCAA
RD_HSPG_3138_R	cloning	CTCGCAGTTCCTCTCGTCCGACCCA
RD_HSPG_3944_F	cloning	GGACCGGCAGCTCTCTCCCCATACC
RD_HSPG_5246_R	cloning	TCCGCCAGTCCCATGAGAAGCTGCT
RD_HSPG_5222_F	cloning	AGCAGCTTCTCATGGGACTGGCGGA
RD_HSPG_6316_R	cloning	GCTGAGTGTGGGTGCTGTGTGTCGC
RD_HSPG_6292_F	cloning	GCGACACACAGCACCCACACTCAGC
RD_HSPG_7292_R	cloning	ATGTTGCGGGAGTTGCTGGCCGAAC
RD_HSPG_7072_F	cloning	CGCATCCCGTACGCCTCCTTGCAAG
RD_HSPG_8130_R	cloning	GTGTTTGGACCAGACGACGGTGGGC
RD_HSPG_8013_F	cloning	GCCACTCATCACGATCACACCGCCG
RD_HSPG_9086_R	cloning	CAGATGTATCGGCCCGCGTCCTCCA
RD_HSPG_8630_F	cloning	AGCTGGATCCGGAGCGTCAAGTGGT
RD_HSPG_9741_R	cloning	GTTGGACAGCGTGGGAAGTGCGAGG
RD_HSPG_9210_F	cloning	CCTTCGCTGCATAGTCCTCGGCCCA
RD_HSPG_10333_R	cloning	CTCCCACGTGCGAACAGTTGAGGCC
RD_HSPG_10264_F	cloning	CCAACCAAGTCCGGCTACACGTGCC
RD_HSPG_11593_R	cloning	GCAGTCCTCCGCTCTCCCCGAACTC
PBT_RGDV_P8_F	Y2H	ATTAACAAGGCCATTACGGCCATGTCGCGCCAAGCTTGGATC
PBT_RGDV_P8_R	Y2H	AACTGATTGGCCGAGGCGGCCCCTTAGTTTACTGTGTAATACCTACC
PBT_RDV_P8_F	Y2H	ATTAACAAGGCCATTACGGCCATGTCACGCCAGATGTGGTTAG

PBT_RDV_P8_R	Y2H	AACTGATTGGCCGAGGCGGCCCCCTAATTTGGTCTATAGTATCTTCCA
pPR3_HSPG_D1_F	Y2H	ATTAACAAGGCCATTACGGCCCAAAGAGTATACCACCGAGTTA
pPR3_HSPG_D1_R	Y2H	TTGACTAAGGCCGAGGCGGCCGTTACAGCAGGTCTCTAATTTCCAG
pPR3_HSPG_D2_F	Y2H	ATTAACAAGGCCATTACGGCCATCGGAGTCCATCCGGTCAG
pPR3_HSPG_D2_R	Y2H	TTGACTAAGGCCGAGGCGGCCGTTATATACAGATCCCGGACACCG
pPR3_HSPG_D3_F	Y2H	ATTAACAAGGCCATTACGGCCAACAACCGAGGTTCCACCTTC
pPR3_HSPG_D3_R	Y2H	TTGACTAAGGCCGAGGCGGCCGTTATGGTGTTCCTGGGGTCGGC
pPR3_HSPG_D4_F	Y2H	ATTAACAAGGCCATTACGGCCATCGAGGCAGGCAAAGACATC
pPR3_HSPG_D4_R	Y2H	TTGACTAAGGCCGAGGCGGCCGTTAGTTGTTGGCGGTGCAGATGT
pPR3_HSPG_D5_F	Y2H	ATTAACAAGGCCATTACGGCCCTGGAGATCACCTTCAAGCC
pPR3_HSPG_D5_R	Y2H	TTGACTAAGGCCGAGGCGGCCGTTAACTGGGACACGGCACCACG
pGEX_HSPG_D3_F	Pull-down	CGGGATCCCGATGAACAACCGAGGTTCCACCTTC
pGEX_HSPG_D3_R	Pull-down	GGAATTCCTTATGGTGTTCCTGGGGTCGGC
RGDV_P8_F	Pull-down/ virus detection	ATGTCGCGCCAAGCTTGGATC
RGDV_P8_R	Pull-down/ virus detection	TTAGTTTACTGTGTAATACCTAC
RD_HSPG_ F	RNAi	ATTCTCTAGAAGCTTAATACGACTCACTATAGGGGGCGTGCGACTGGTGTACTA
RD_HSPG_R	RNAi	ATTCTCTAGAAGCTTAATACGACTCACTATAGGGGAGTGTGAAGTGGTGCGTCTTG
GFP_F	RNAi	ATTCTCTAGAAGCTTAATACGACTCACTATAGGGAAGTTCAGCGTGTCCGGCGA
GFP_R	RNAi	ATTCTCTAGAAGCTTAATACGACTCACTATAGGGGAAGTTCACCTTGATGCCGTT

Supplementary References

1. Chen, Y. et al. Adverse effects of *Rice gall dwarf virus* upon its insect vector *Recilia dorsalis* (Hemiptera: Cicadellidae). Plant Dis. **100**, 784-790 (2016).
2. Wang, H., Xu, D., Pu, L. & Zhou, G. *Southern rice black-streaked dwarf virus* alters insect vectors' host orientation preferences to enhance spread and increase *Rice ragged stunt virus* co-infection. Phytopathology **104**, 196-201 (2014).
